# Novel method for transaxillary Impella 5.0 implantation in patients with tortuous arterial anatomy

**DOI:** 10.1002/ccr3.4186

**Published:** 2021-07-19

**Authors:** Bryant Fisher, Conrad Smith, Ibrahim Sultan, Arman Kilic

**Affiliations:** ^1^ Division of Cardiac Surgery University of Pittsburgh Medical Center Pittsburgh PA USA

**Keywords:** cardiogenic shock, cardiothoracic surgery, impella, mechanical support

## Abstract

A novel method of transaxillary Impella 5.0 implantation utilizing a vascular sheath can overcome difficult arterial anatomy and provide mechanical support for patients in critical cardiogenic shock.

## Graphical abstract

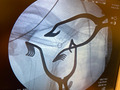

## 1 INTRODUCTION

An axillary approach to Impella 5.0 implantation is becoming more common given its advantages over femoral placement. In this case report, we detail a technical approach utilizing a large‐bore vascular sheath to dilate and straighten difficult arterial segments to facilitate insertion of the Impella 5.0 device.

Cardiogenic shock is a condition that occurs when the heart is unable to perfuse the rest of the body resulting in significant end‐organ dysfunction and mortality. It arises when a physiologic insult damages the myocardium to such an extent that contractile function is impaired. It is often necessary to support the heart utilizing various temporary and durable mechanical support devices to prevent the sequelae associated with this condition.

## 2 CASE REPORT

A 50‐year‐old male patient with a history of hypertension and diabetes experienced an out‐of‐hospital arrest in the setting of severe chest pain. He underwent numerous defibrillations in the field for ventricular tachycardia before return of spontaneous circulation. The patient was found to have an ST elevation myocardial infarction and was taken emergently for stenting of his left anterior descending artery. Following the procedure, he was persistently hypotensive requiring placement of an intra‐aortic balloon pump and initiation of multiple vasoactive medications including epinephrine, norepinephrine, and dopamine. Given his elevated filling pressures and persistent cardiogenic shock, the decision was made to proceed to the OR for right transaxillary Impella 5.0 ventricular assist device placement. Due to the urgent nature of the procedure, a computed tomography scan of his chest was not obtained.

**FIGURE 1:**
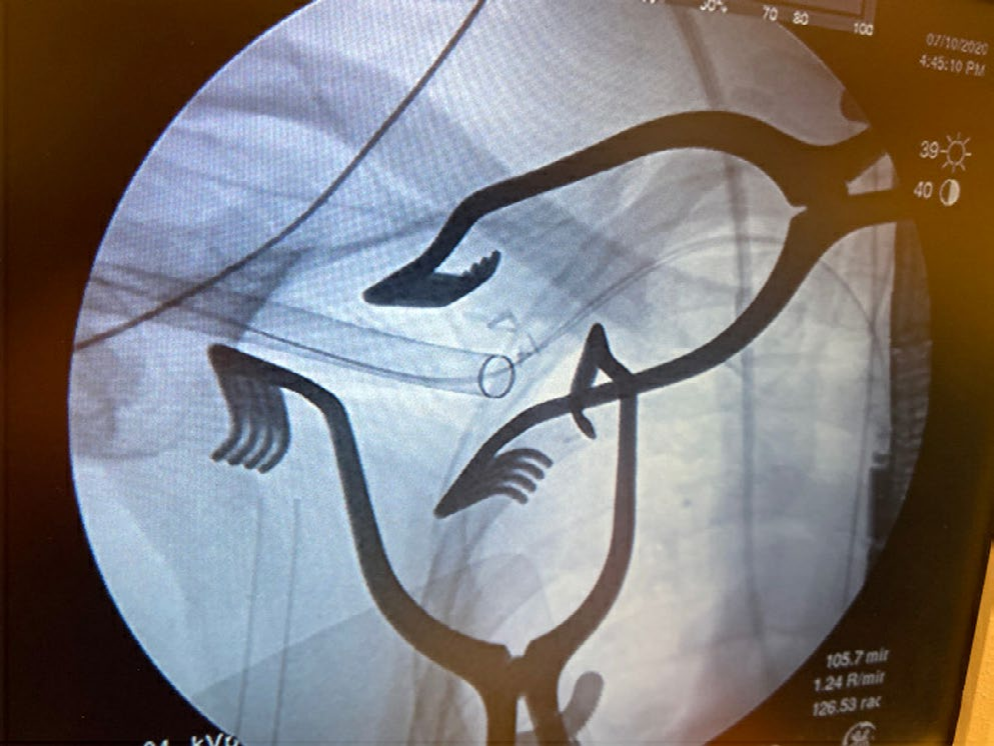
Fluoroscopic view of the drysheath advanced into the axillary artery over a stiff wire to straighten the angulation in the axillary artery

**FIGURE 2:**
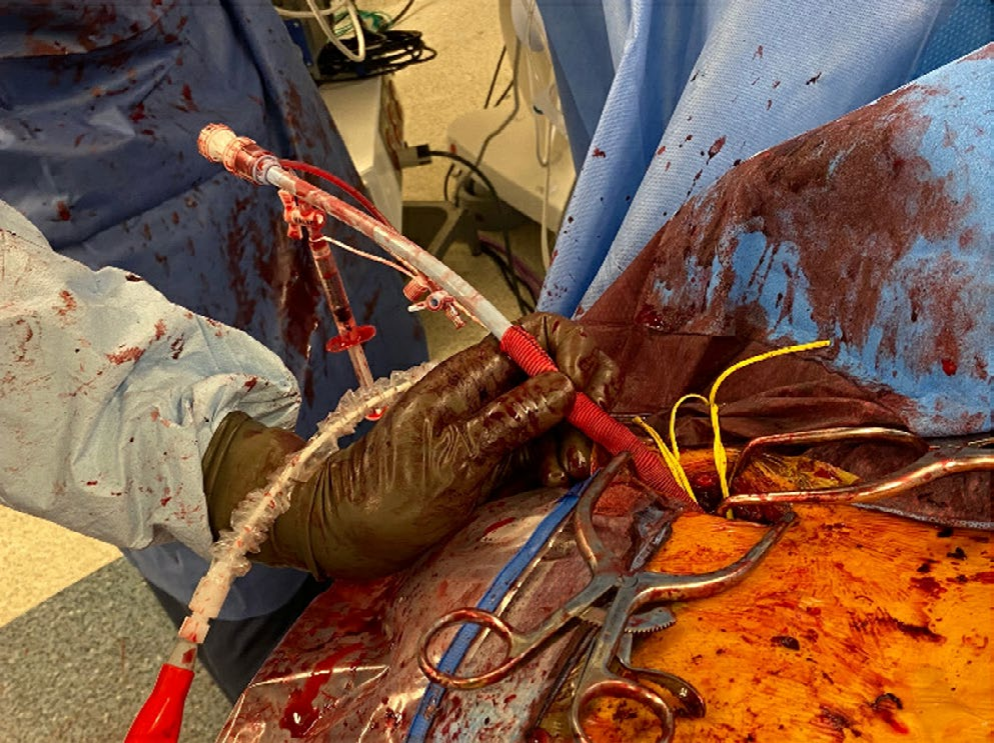
The dry sheath is advanced through the dacron graft and left in place to be used as a conduit for the Impella 5.0

**FIGURE 3:**
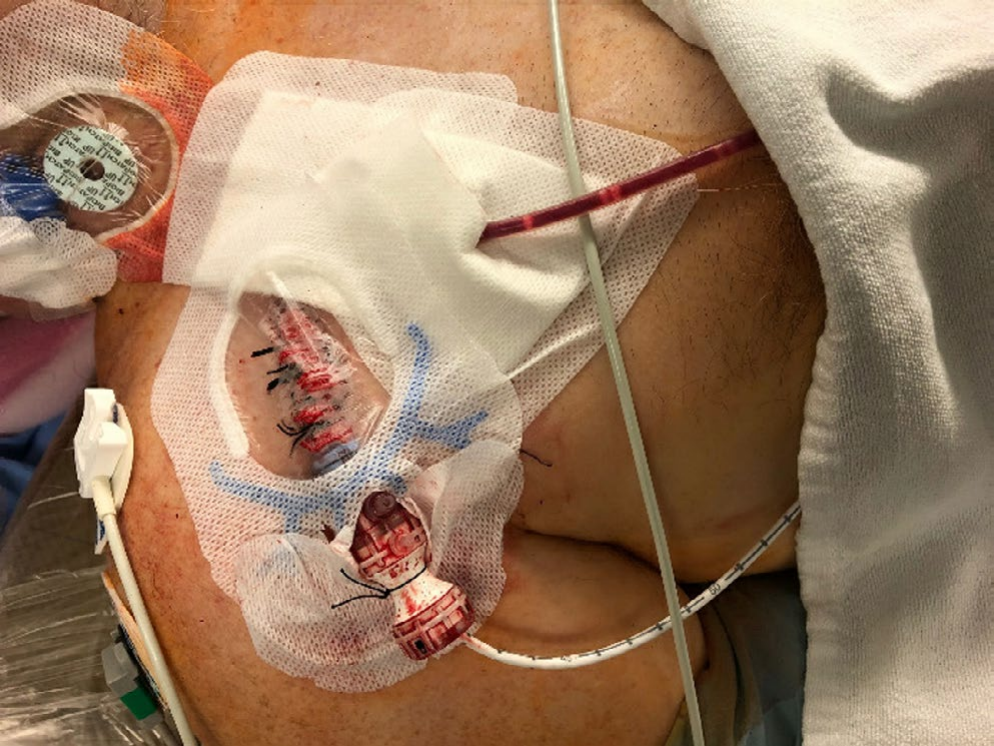
Final configuration of the Impella 5.0 secured in place within the 24 French drysheath

An axillary cutdown with a 4 cm subclavicular incision was performed. After obtaining proximal and distal control, an arteriotomy was made and a 10 mm Dacron graft was sewn in an end‐to‐side fashion onto the axillary artery. A peripheral guidewire was inserted through a standard Judkins catheter across the aortic valve. After insertion of the device through the Dacron graft, difficulty was encountered advancing the 21‐French motor housing through an angulation within the artery. The decision was made to proceed utilizing a long exchange wire and a 0.018‐in guidewire to position a 24‐French dryseal sheath past the angulation. The Impella 5.0 device was then advanced over a stiff wire through the diaphragm of the sheath and across the aortic valve with fluoroscopic guidance. The device was secured with a short segment of the dryseal system in place at the level of the skin, and adequate flows were obtained (Figures 1, 2, 3). Patient was successfully supported with the Impella 5.0 device for 3 days with improvement in end‐organ perfusion. On postoperative day 3, patient had a massive thromboembolic stroke despite therapeutic anticoagulation on a heparin drip. Care was withdrawn due to the massive neurologic injury that was sustained.

The second patient is a 63‐year‐old female patient with history of rheumatoid arthritis, lupus, and nonischemic cardiomyopathy (baseline ejection fraction of 15%‐20%) who presented in acute decompensated heart failure. Despite initiation of dopamine, the patient persisted in a low‐output state. The decision was made to pursue Impella 5.0 placement as a bridge‐to‐transplant.

The operation proceeded in a similar manner with an axillary artery cutdown and end‐to‐side anastomosis of a 10 mm Dacron graft. The Impella 5.0 device was unable to be advanced due to tortuosity in the axillary artery. A 22‐French glide sheath was placed through the graft over a wire and advanced into the aorta under fluoroscopic guidance. The Impella was passed through the sheath across the aortic valve without difficulty, and adequate flows were obtained. The sheath and device were left in place and secured. Ultimately, the patient was supported on the device without complication and underwent successful heart transplant 2 weeks following Impella 5.0 placement.

## 3 DISCUSSION

The Impella 5.0 device has several advantages that have led us and other groups to favor it as a temporary mechanical circulatory support bridging modality in patients presenting with cardiogenic shock. Foremost, it is sternal‐sparing and can therefore avoid patient debilitation, infection risk, and significant blood transfusions. A transaxillary approach also avoids the femoral vessels, thereby allowing mobility and limiting deconditioning. Rates of hemolysis are low, and the device is typically well tolerated for several weeks if not months. Finally, the device is able to provide 5 L of flow and left ventricular unloading which can more rapidly reverse endo‐organ dysfunction and promote myocardial recovery.

The utilization of large‐bore sheaths to address angulation and tortuosity of the axillary artery for device insertion can allow the use of this ventricular assist device in a broader range of patients. Although a preoperative computed tomography study is useful in assessing tortuosity and size of the axillary artery, we do not routinely obtain it as most patients are presenting in cardiogenic shock and decompensated heart failure. In addition, most axillary arteries are distensible, and it is rarely required to abort a transaxillary approach for this procedure.

A retrospective review by Tarabichi et al details the various indications and complications associated with transaxillary insertion of the Impella 5.0. Primary reasons for insertion include upgrade of support from an intra‐aortic balloon pump (IABP), amelioration of left ventricular distention on extracorporeal membrane oxygenation (ECMO), and long‐term support as a bridge‐to‐transplant or bridge‐to‐therapy. Of the 40 examined, 23 patients already had devices in place via femoral access including IABP and Impella CP while nine patients were maintained on venoarterial ECMO. Given the limited access options, the transaxillary route with the Impella 5.0 is a reasonable and safe alternative to provide upgraded access to these patients. It is also our preferred approach regardless of other existing devices due to the reasons highlighted above. Of note, the complications associated with this approach predictably included right arm ischemia and neuropathy in three patients. The mean duration of support on the Impella device was 21 days, and 17 patients underwent further surgical intervention (heart transplant, durable mechanical support). This case series demonstrates the utility and feasibility of Impella 5.0 placement via an axillary cutdown.^1^

Bertoldi et al^2^ detailed the use of the Impella 5.0 device to wean patients from venoarterial ECMO as a bridge to durable LVAD placement. Another retrospective review examined its utility in patients with acute‐on‐chronic heart failure. They noted improved end‐organ function, improved left ventricular unloading, and reduced need for anticoagulation^3^ Esposito et al^4^ highlight the potential survival benefits of early mobility in patients with axillary Impella 5.0 placement.

There are very few reports detailing technical troubleshooting in transaxillary insertion of the Impella 5.0 device. In their report, Shah et al^5^ detail a similar method employing a vascular sheath to overcome tortuous arterial anatomy during this procedure. With increasing popularity of this support platform and approach for bridging to durable support or recovery, techniques such as those described will hopefully be of value in navigating otherwise prohibitive anatomical issues.

## CONFLICT OF INTEREST

None declared.

## AUTHOR CONTRIBUTIONS

BF: prepared and wrote the manuscript with assistance from CS, IS, and AK. AK and CS: participated in the case described in the report and provided the necessary details and technical guidance. All authors participated in editing and revision of the report.

## DISCLOSURES

None.

## INFORMED CONSENT

Not applicable to case report. Patients are de‐identified in the body of the text.

## Data Availability

Not applicable. No data are associated with this report.
